# Modified Hyperbranched Polyglycerol as Dispersant for Size Control and Stabilization of Gold Nanoparticles in Hydrocarbons

**DOI:** 10.1186/s11671-017-2296-1

**Published:** 2017-09-06

**Authors:** Yanyu Shen, Guijin He, Yongsheng Guo, Hujun Xie, Wenjun Fang

**Affiliations:** 1 Department of Chemistry, Zhejiang University, Hangzhou, 310058 China; 2 Department of Applied Chemistry, Zhejiang Gongshang University, Hangzhou, 310018 China

**Keywords:** Hyperbranched polyglycerol, Gold nanoparticles, Hydrocarbons, Nanofluids, Dispersion stability

## Abstract

Hyperbranched polyglycerol (HPG) is modified with dodecanethiol (DS) via the “thiol−ene” click reaction to obtain an amphiphilic product DSHPG. The molecular structures of DSHPG samples are characterized by NMR, FTIR, and GPC, and the thermal behaviors are characterized by DSC and TGA. Gold nanoparticles (Au NPs) are prepared with DSHPG as the stabilizer and surface-modification reagent. The size of Au NPs can be tuned by changing the molecular weight of HPG. It is observed that the HPG molecular weights of 1123, 3826, and 55,075 lead to the NP diameters of 4.1 nm for Au@DSHPG-1, 9.7 nm for Au@DSHPG-2, and 15.1 nm for Au@DSHPG-3, respectively. The morphology and size of Au NPs are characterized by TEM and DLS. Especially, the dispersion abilities of Au NPs in different pure solvents and co-solvent mixtures are investigated. The long alkyl chains on DSHPG give the ability of Au NPs to be well dispersed in nonpolar solvents. Hydrocarbon-based nanofluids can be obtained from the hydrophobic Au NPs dispersed into a series of hydrocarbons. The dispersion stability for Au NPs in hydrocarbons is monitored by UV-Vis spectroscopy, and the relative concentration of Au NPs is observed to still maintain over 80% after 3600 h.

## Background

Because of the high energy density and high heat sink, endothermic hydrocarbon fuels have played an important role in the aerospace field. They can be used as flammable coolants in the engine to provide regenerative cooling effects to the aircrafts. Catalytic cracking is an effective approach to improve the endothermic capability of hydrocarbon fuels [1–3], and noble metal NPs are usually used as the catalysts [4, 5]. Gordon firstly proposed the idea of adding metal powders into conventional fuels [6]. Zhang found Pt and Pd NPs could markedly enhance the cracking of JP-10, and Pt NPs could lower the onset temperature of the cracking reaction from 650 to 600 °C [7]. Yue applied Pd NPs in decalin and aviation kerosene, and the conversion, gas yield, and heat sink of these hydrocarbon fuels were increased significantly [8, 9]. In our previous work, the catalytic activity of the Au NPs dispersed in JP-10 as a pseudohomogeneous system was also investigated, and the conversion of JP-10 catalyzed by Au NPs was found to be higher than that of pure JP-10 from thermal cracking [10]. However, the acquisition of a stable solid-liquid suspension is still a major challenge to prepare the nanoparticle-contained fuels [11].

As a practical technique, researchers have come up with the coating method to give the NPs with a protective layer [12]. Hyperbranched polymer is considered as a kind of effective coating material. The globular macromolecules with unique structure and properties can offer an excellent platform for the synthesis and stabilization of metal NPs [13, 14]. Hyperbranched polyglycerol (HPG) has a hyperbranched polyether “core” and is terminated with hydroxyl groups, which provides perfect medium for hosting and stabilizing metal NPs [15–17]. The HPG-stabilizing approach can be used to prepare a wide variety of inorganic nanocrystals with good stability and biocompatibility [18]. Furthermore, HPG has been developed as promising initiators to promote the cracking of hydrocarbon fuels [19, 20]. The palmitoyl chloride-modified HPG (PHPG) was applied as a “radical package” for hydrocarbon fuels, and significant promotion effects on the conversion, gas yield, and heat sink of tridecane and kerosene with the presence of PHPG were observed [21].

HPG with thioether structures may provide long-term stability for metal NPs since sulfur is a universal coordinate site for soft metal ions compared with oxygen-containing shells, such as esters and acetyls [22–24]. The thiol−ene click chemistry is an effective method to introduce thioether structure, which contains kinds of highly selective, simple orthogonal reactions with high efficiency under mild conditions [25]. Thiol–ene systems polymerized by a free-radical chain mechanism can result in the addition of a thiol group to the double bond [26]. Unfortunately, only several examples of thioether-substituted hyperbranched polymers have been reported to date [16].

In this work, the amphiphilic HPG with thioether structures (DSHPG) is synthesized by the click chemistry of modified HPG and 1-dodecanethiol. The long alkyl chains give DSHPG good solubility in hydrocarbon fuels. DSHPG is used as surface-modification reagent for Au NPs to obtain well-dispersed Au@DSHPG. The relationship between the size of Au NPs and the molecular weights of HPG is systematically investigated. The dispersion stability of Au NPs in different pure solvents and co-solvent mixture is also examined.

## Methods

### Materials

**Scheme 1:**
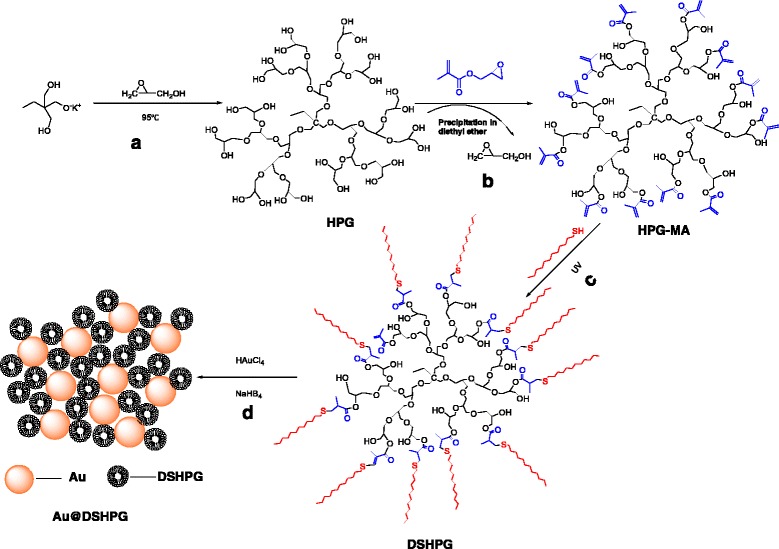
Preparation of HPG, HPG-MA, DSHPG, and Au@DSHPG

(±)Glycidol (96%), potassium methylate (95%), 1,1,1-tris(hydroxymethyl)propane (TMP, 98%), 4-(*N*,*N′*-dimethyl amino)pyridine (99%), 1,4-dioxane (99%), glycidyl methacrylate (97%), benzophenone (99%), chloroauric acid (48–50%, Au basic), tetraoctylammonium bromide (98%), and sodium borohydride (98%) were purchased from Aladdin Chemical Reagent Corporation (Shanghai, China). Toluene (99.5%), methanol (99.5%), chloroform 99%), diethyl ether (99.7%), dichloromethane (99.5%), dimethyl sulfoxide (99%), toluene (99.5%), hexane (97%), cyclohexane (97%), decahydronaphthalene (98%), 1-dodecane (98%), and octane (98%) were purchased from Sinopharm Chemical Reagent Corporation (Jiangsu, China). 1-Tetradecane was purchased from Shanghai Xin Ran chemical technology corporation. The samples of HPG, HPG-MA, DSHPG, and Au@DSHPG were prepared in this laboratory according to the procedure shown in Scheme 1.

### Preparation of HPG

HPG was prepared via a ring-opening anionic polymerization method described by previously [27]. 1,1,1-Tris(hydroxymethyl) propane (TMP, 0.278 g, 1.5 mmol) was partially deprotonated with 0.1 mL of a potassium methylate solution (1.25 mmol of CH_3_OK in methanol), followed by distillation of excess methanol from the melt. Then, glycerol (50 mL, purified by vacuum distillation) was added dropwise at 95 °C over a period of 12 h using a syringe pump. The product was neutralized by filtration over a cation-exchange resin and precipitated twice in acetone. Finally, HPG was dried for 24 h at 85 °C in vacuum. The molecular weights of HPG were controlled by the monomer/initiator ratio and the addition of 1,4-dioxane into the melt (Scheme 1a).

### Preparation of Methacrylated HPG-MA

The synthesis of HPG-MA was carried out on the basis of the procedure described by Marion [28]. HPG (1 g) and 4-(*N*, *N′*-dimethyl amino) pyridine (2 g) were dissolved in dimethyl sulfoxide (9 mL) at room temperature under a nitrogen atmosphere. Glycidyl methacrylate (10 mL) was then added dropwise. After 24 h of stirring at room temperature, HPG-MA was precipitated in diethyl ether and subsequently dried overnight (Scheme 1b).

### Preparation of DSHPG by Click Chemistry

Dried HPG-MA (4 mL) and chloroform (4 mL) were added in a tubular photo reactor. After dissolution of HPG-MA, 1-dodecanethiol (2 mL) and appropriate amount of benzophenone were added. After stirring for 1 h under ultraviolet ray, the polymer was purified by chloroform dialysis and dried at room temperature (Scheme 1c).

### Preparation of Au@DSHPG

Au NPs were prepared by the following procedure. HAuCl_4_ aqueous solution (20 mL, 0.59 mmol) was mixed with tetraoctylammonium bromide (0.42 g) in toluene (120 mL). The mixture was stirred vigorously, and the organic layer was collected. Then, DSHPG (0.2 g) was added. After that, the solution of fresh NaBH_4_ (0.15 g, 3.97 mmol) in water (20 mL) was added dropwise, and the mixture was kept stirring for 0.5 h. The mixture was allowed to settle for 2 days to ensure the complete transfer of gold to enter into the amphiphilic polymer. The precipitate was discarded, and the clear solution was subjected to rotary evaporation to remove toluene. Finally, the brownish solid was dried under vacuum at room temperature (Scheme 1d).

### Characterization of HPG, HPG-MA, DSHPG, and Au@DSHPG

Nuclear magnetic resonance (NMR) spectra of HPG, HPG-MA, and DSHPG were acquired on a Bruker Advance 400 spectrometer in *d*_4_-methanol, *d*_6_-dimethylsulfoxide, and *d*-chloroform, respectively. The spectra were referenced to the residual signals of the deuterated solvents. Fourier Transform Infrared (FTIR) spectra were recorded on a Nicolet iS10 spectrometer from Thermo Fisher Scientific Corp., (Film or KBr disk).

Number-average molecular weight ($\overline{M_n}$) and its distribution for HPG samples were determined on a gel permeation chromatography (GPC) (Waters, USA) with distilled water as the eluent at the flow rate of 1.0 mL min^− 1^. The system was calibrated by using linear poly (methyl methacrylate) standards with narrow size distribution. Each HPG aqueous solution was prepared at the concentration of 1.0 mg mL^− 1^.

Differential scanning calorimetry (DSC) measurements of HPG, HPG-MA, DSHPG, and Au@DSHPG-2 were performed on a Q2000 differential scanning calorimeter (TA Instruments, USA). The temperature range was set from − 70 to 100 °C (HPG) or − 70 to 150 °C (HPG-MA, DSHPG and Au@DSHPG-2) under the scanning rate of 3.0 °C min^− 1^. Thermo-gravimetric analyses (TGA) of HPG and Au@DSHPG-2 were performed on a Q50 Thermogravimetric Analyzer (TA Instruments, USA). The temperature range was set from room temperature to 600 °C under nitrogen atmosphere with the heating rate of 10 °C min^− 1^.

The morphology of Au@DSHPG was observed by employing transmission electron microscopy (TEM, CM-200, PHILIPS). The average sizes were monitored by dynamic light scattering (DLS, ZEN 3600, Malvern Instruments). The stability of Au nanofluids was evaluated by the optical absorption, which was detected by a UV−Vis spectrometer (Shimadzu, Japan, UV-1750) equipped with 1.0 cm quartz cells.

## Results and Discussion

### Molecular Structures of HPG, HPG-MA, and DSHPG

**Fig. 1:**
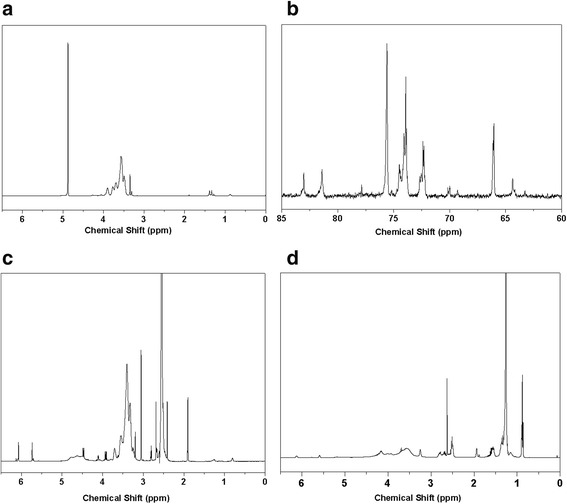
NMR characterizations of HPG, HPG-MA, and DSHPG **a** ^1^H NMR spectrum of HPG, **b** ^13^C NMR spectrum of HPG, **c** ^1^H NMR spectrum of HPG-MA, **d** ^1^H NMR spectrum of DSHPG

The ^1^H and ^13^C NMR spectra of HPG, HPG-MA, and DSHPG are shown in Fig. 1. The peaks between 3.3 and 3.9 ppm in the ^1^H NMR spectrum of HPG (Fig. 1a) are the signals of hydrogens of methyl, methylene, and methine. The ^13^C NMR of HPG (Fig. 1b) is consistent with that in the literature [29]. In the ^1^H NMR spectrum of HPG-MA (Fig. 1c), the signals of hydrogens of methacryloyl groups appear at 1.8 ppm, and the hydrogens connected to the C=C bond appear at 5.6 and 6.0 ppm. After the click reaction, the signals for the hydrogens connected to the C=C bond are significantly reduced, and those for dodecyl group appear from 0.8 to 2.0 ppm (Fig. 1d).

**Fig. 2:**
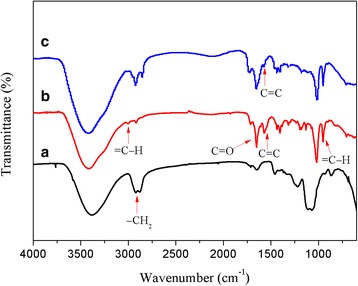
FTIR spectra of **a** HPG, **b** HPG-MA, and **c** DSHPG

In the Fourier Transform Infrared (FTIR) spectra showed in Fig. 2, a strong absorption band of C=C stretching vibration peak apparently appears at 1562 cm^− 1^ after the esterification of HPG, and the bands of =C−H and C=O are observed at 3003 cm^− 1^ and 1652 cm^− 1^, respectively. The signals of double bond groups are significantly reduced in the FTIR spectrum for the click reaction product DSHPG. The structural characterizations indicate that successful esterification and S-alkylation of HPG are carried out and then the products of HPG-MA and DSHPG are obtained.

### Thermal Behaviors of HPG, HPG-MA, DSHPG, and Au@DSHPG

**Fig. 3:**
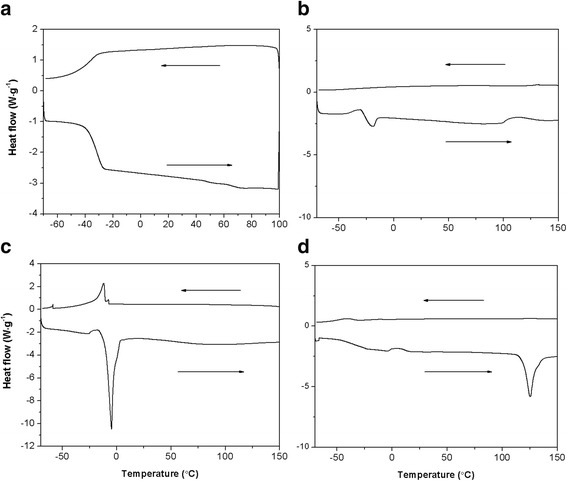
DSC curves for **a** HPG, **b** HPG-MA, **c** DSHPG, and **d** Au@DSHPG-2 at the scanning rate of 3 °C min^− 1^

DSC results of HPG, HPG-MA, DSHPG, and Au@DSHPG-2 are shown in Fig. 3. The temperature of glass-transition point (*T*_g_) of HPG can be observed at − 37.5 °C (Fig. 3a), which corresponds to polymer chain segment movement of the “frozen” or “thaw” process achieved by the motion of single bonds in the main chain. The crosslinking point of HPG-MA is determined to be 110 °C, and the value of *T*_g_ is − 20.8 °C (Fig. 3b). It indicates that the factors that can affect polymer chain flexibility have influences on *T*_g_. The crosslinking of C=C bond in HPG-MA limits the chain segment motion, and the *T*_g_ value is much higher than that of HPG. Since the long alkyl chains on DSHPG are well aligned, DSHPG is apt to crystallize. A melting peak at − 4.7 °C and a cold crystallization peak at − 12.34 °C can be observed for DSHPG (Fig. 3c). However, these peaks are no longer observed for Au@DSHPG (Fig. 3d). It means that the introduction of Au NPs in Au@DSHPG probably leads to the collapse of polymer chain and destructs the neat arrangement of molecular chain.

**Fig. 4:**
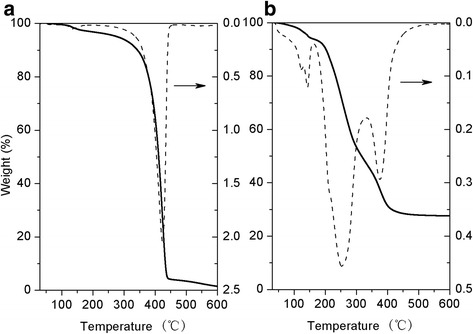
TGA and DTG curves for **a** HPG and **b** Au@DSHPG-2 at the heating rate of 10 °C min^− 1^

The thermal stability for HPG and Au@DSHPG is detected by TGA, with the results shown in Fig. 4. It indicates that the cracking of HPG core occurs around 425 °C (Fig. 4a). Hence, for the cracking of Au@DSHPG (Fig. 4b), the weight loss peak at 400 °C is attributed to the cracking of HPG, and other weight loss peaks should be attributed to the cracking of the polymer shell of DSHPG. The Au content of the Au@DSHPG-2 sample is 27.2 wt%.

### Size Control of Au@DSHPG

A series of HPG samples are obtained with the $\overline{M_n}$ values varied from 1123 to 57,000 (Table 1). It is inferred that a larger monomer (glycerol)/initiator (TMP) ratio can generate a higher molecular weight for the polymer. Moreover, the molecular weight of the obtained polymer increases to a much higher level in the presence of 1,4-dioxane, which takes the action of an emulsifier and can create a micro-environment that benefits the cation exchange to protect the anionic polymerization [30].

**Table 1:** Molecular weights and average particle sizes of HPG samples

HPG	Glycerol (mL)	TMP (mmol)	Dioxane (mL)	$\overline{M_n}$	$\overline{d_n}$ (nm)
HPG-1	30	1.5	0	1123	2.68
HPG-2	30	1.5	10	2733	3.12
HPG-3	50	1.5	0	2400	2.50
HPG-4	50	1.5	20	39,400	10.23
HPG-5	60	1.5	10	55,075	11.24
HPG-6	60	1.5	20	57,000	12.07
HPG-7	70	1.5	0	3826	2.82

**Fig. 5:**
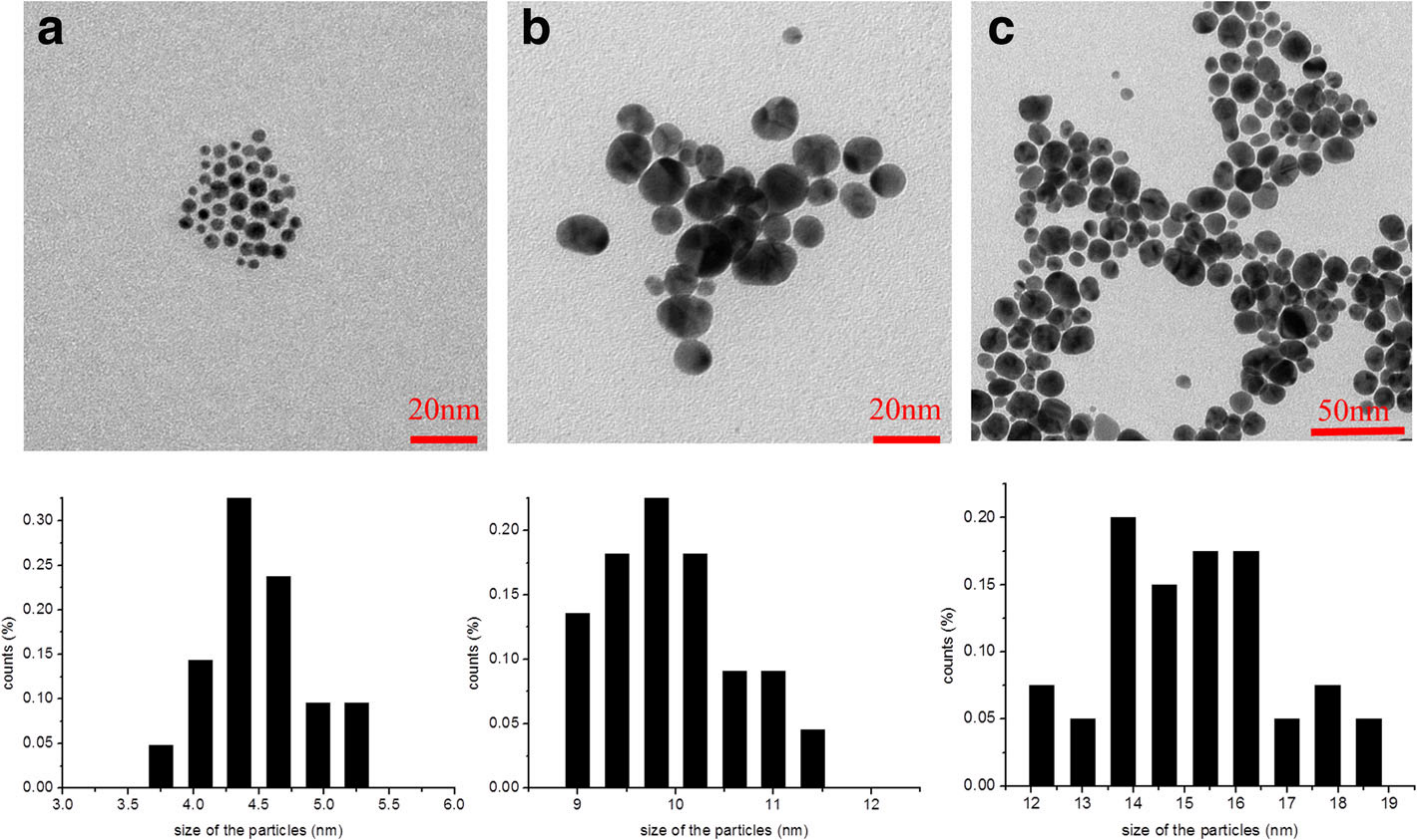
TEM image and size distribution of Au@DSHPG. **a** Au@DSHPG-1 (HPG $\overline{M_n}$ = 1123), **b** Au@DSHPG-2 (HPG $\overline{M_n}$ = 3826), and **c** Au@DSHPG-3 (HPG $\overline{M_n}$ = 55,075)

The results of dynamic light scattering (DLS) indicate that the size of HPG in water increases with the increase of HPG molecular weight. On the basis of different sizes or molecular weights of the core HPG molecules, the size control of Au NPs is then performed by dodecanethiol-modified HPG (DSHPG). The TEM images of Au@DSHPG are shown in Fig. 5.

The sizes of 4.1, 9.7, and 15.1 nm for Au@DSHPG-1, Au@DSHPG-2, and Au@DSHPG-3 are observed corresponding to the $\overline{M_n}$ values of 1123, 3826, and 55,075 for HPG, respectively. Clearly, the size of Au NPs is strongly affected by the size of the HPG core. There is a positive relationship between the molecular weight of HPG and the size of Au NPs. The HPG with higher molecular weight possesses bigger polyether “core” and can result in the formation of bigger-sized NPs. From the observed sizes of HPG and NPs, it can be speculated that several macromolecules of HPG, other than just one, take effects on the stabilization of one nanoparticle. The smaller HPG molecules have a better protection (or separation) effect against the contact and fusion of different nanoparticles.

### Dispersion Ability of Au@DSHPG in Different Solvents

**Fig. 6:**
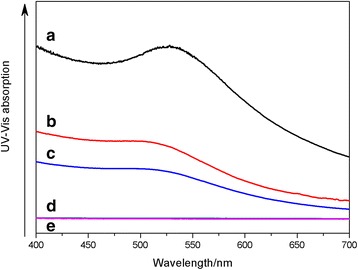
UV-Vis absorption curves for saturated solutions of Au@DSHPG-2. **a** Methylbenzene (diluted 100 times), **b** diethyl ether (diluted six times), **c** ethyl acetate, **d** acetonitrile, and **e** water

UV-Vis spectrophotometry is employed to investigate the dispersion ability of Au@DSHPG in different pure solvents and co-solvent mixtures. Au@DSHPG-2 is dispersed in several pure solvents with different polarities, such as toluene, diethyl ether, ethyl acetate, acetonitrile, and water. The UV-Vis spectra of the saturated solutions of Au@DSHPG-2 are presented in Fig. 6. Since the absorption values of the saturated solutions of Au NPs in toluene and diethyl ether are too high to detect, the systems are diluted with the solvents for UV−Vis measurements. It is illustrated that the dispersion abilities of Au NPs stabilized with DSHPG in nonpolar solvents are significantly better than those in polar solvents due to the nonpolar alkyl chain of DSHPG.

**Fig. 7:**
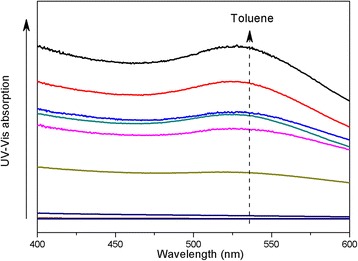
UV-Vis absorption curves for saturated solutions of Au@DSHPG-2 in different mixtures of toluene and acetonitrile. The dashed arrow indicates the increasing volume fraction of toluene from 0 to 1 with 0.1 interval

**Fig. 8:**
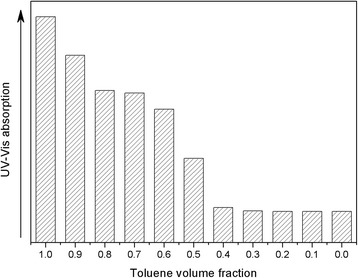
UV-Vis absorption at 521 nm of saturated solutions of Au@DSHPG-2 in different mixtures of toluene and acetonitrile

To further reveal the relationship between the dispersion ability of Au@DSHPG and the solvent polarity, a series of Au@DSHPG-2-containing solutions in mixtures of toluene and acetonitrile are prepared. The polarities are tuned by the ratios of two components. The UV-Vis absorption curves for these solutions are shown in Fig. 7. The dispersion ability of Au NPs is gradually enhanced when the fraction of toluene is increased. The absorption values at 521 nm of saturated solutions in different mixtures of toluene and acetonitrile are shown in Fig. 8. The results show that the weaker the polarity of the solvent, the more feasible dispersion of Au@DSHPG.

The dissolution process can be evaluated theoretically from the solubility coefficient. For the polymer DSHPG, the solubility coefficient can be calculated with the following equation [31]:

1 $$ \delta =\frac{\sum F}{\overline{V}}=\frac{\rho \sum G}{\overline{M}} $$

where *ρ* is the density of DSHPG, $\overline{M}$ is the molar mass of repetitive unit in DSHPG, and *G* is the molar gravitational constant.

A smaller difference between *δ*_1_ and *δ*_2_ results in a better miscibility of two liquids. The detailed data of *δ*_1_ (solvent) [32] and *δ*_2_ (DSHPG) at 25 °C and 101.3 kPa are listed in Table 2. It is noted that the solubility coefficient of toluene is the closest to that of DSHPG, followed by ethyl acetate, diethyl ether, acetonitrile, and water. This means the dissolution process becomes more difficult under room temperature when increasing the polarity of solvents. The results from theoretical calculation are consistent with those from UV-Vis analyses. It proves that DSHPG successfully endows the Au NPs with lipophilicity and hydrophobicity, making it possible for the application of Au@DSHPG in hydrocarbons.

**Table 2:** Solubility coefficients of DSHPG and different solvents

Solvent	*δ*_1_/cal^1/2^ cm^− 3/2^	*δ*_2_/cal^1/2^ cm^− 3/2^
Toluene	8.90	8.93
Diethyl ether	7.40	8.93
Ethyl acetate	9.10	8.93
Acetonitrile	11.90	8.93
Water	23.20	8.93

### The Stability of Au@DSHPG in Hydrocarbons

**Fig. 9:**
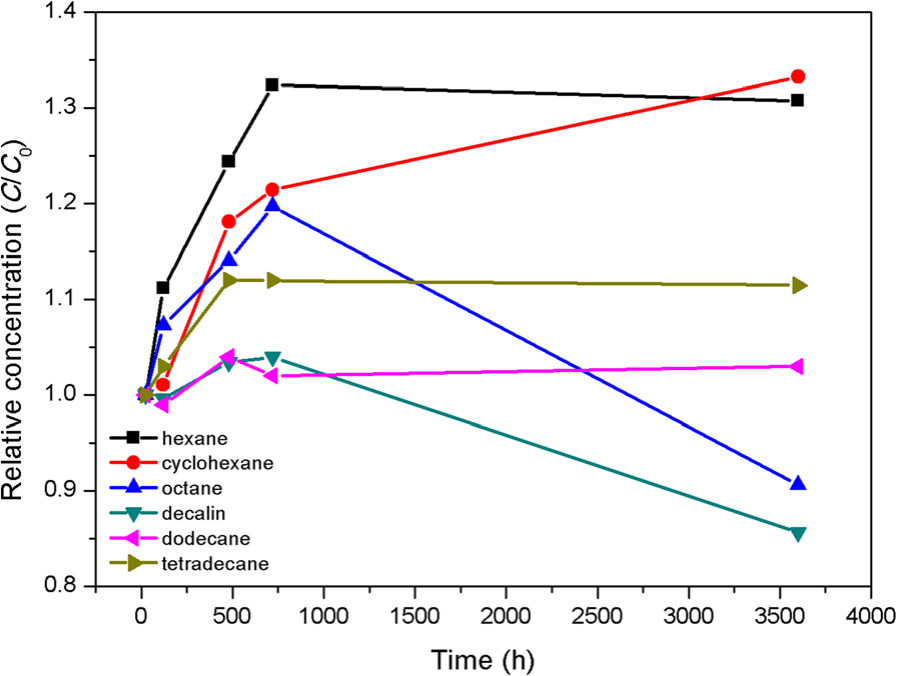
Relative supernatant particle concentration of Au@DSHPG-2 in different hydrocarbons

Generally, there is a linear relation between the supernatant concentration and the absorbance of suspended particles according to the Bouguer-Lambert-Beer law [33]. To reveal the relative stability of nanofluids, Au@DSHPG-2 is dispersed in hexane, cyclohexane, octane, decalin, dodecane, and tetradecane, and the UV-Vis absorption against sediment time is investigated. The change of absorption of Au NPs versus settling time in these hydrocarbon fuels is shown in Fig. 9. Through the DSHPG modification, Au NPs are surrounded by the network of hydrophilic hyperbranched polymer DSHPG, which weakened the absorption of Au NPs. When Au@DSHPG is mixed with hydrocarbons, the hydrocarbon molecules slowly permeate into the polymer, the polymer goes swelling until dissolved, and the Au NPs are gradually released from the bulky molecules, leading to the increase of the absorption. This process is rather time-consuming under ambient conditions, which takes hundreds of hours for the Au NPs to be completely exposed. As shown in Fig. 9, the relative concentrations of NPs in different solvents are increased during the period of 0~750 h. With the further increase of sediment time from 750 to 3600 h, the relative concentrations of Au NPs in hexane, octane, and decalin almost keep constant. However, those slightly drop in dodecane and tetradecane. After the systems have been kept for 3600 h, the relative concentrations of Au NPs are still maintained over 80%. Therefore, it can be proposed that the modification with DSHPG enables the Au NPs to possess long-term storage stability in hydrocarbon-based fuels.

## Conclusions

A series of hyperbranched polyglycerol (HPG) with different number-average molecular weights have been prepared. They are successfully employed to prepare the dodecanethiol-modified HPG (DSHPG) and DSHPG-modified Au NPs (Au@DSHPG). The size of Au@DSHPG can be controlled by adjusting the molecular weight of HPG, and the higher molecular weight of HPG leads to the bigger size of Au NPs. UV-Vis observation results show that the dispersion abilities of the Au@DSHPG in nonpolar solvents (such as toluene and diethyl ether) are significantly better than those in polar solvents (such as water and acetonitrile), which proves the strong effect of DSHPG on the dispersion of Au NPs in solvents. Sedimentation measurement results indicate the Au@DSHPG can be stabilized for more than 3600 h without significant deposition in hydrocarbons. Such kinds of hydrocarbon-based nanofluids may have the potential to reach higher heat sink values during their thermal cracking processes due to the catalytic effects of Au NPs.

## Abbreviations

Au@DSHPG-1: Gold nanoparticles stabilized by DSHPG with a diameter of 4.1 nm
Au@DSHPG-2: Gold nanoparticles stabilized by DSHPG with a diameter of 9.7 nm
Au@DSHPG-3: Gold nanoparticles stabilized by DSHPG with a diameter of 15.1 nm
DS: Dodecanethiol
DSHPG: Hyperbranched polyglycerol (HPG) modified with dodecanethiol (DS)
HPG: Hyperbranched polyglycerol
